# Polymorphisms of rs2483205 and rs562556 in the PCSK9 gene are associated with coronary artery disease and cardiovascular risk factors

**DOI:** 10.1038/s41598-021-90975-0

**Published:** 2021-06-01

**Authors:** Min-Tao Gai, Dilare Adi, Xiao-Cui Chen, Fen Liu, Xiang Xie, Yi-Ning Yang, Xiao-Ming Gao, Xiang Ma, Zhen-Yan Fu, Yi-Tong Ma, Bang-dang Chen

**Affiliations:** 1 Xinjiang Key Laboratory of Cardiovascular Disease, Clinical Medical Research Institute, First Affiliated Hospital of Xinjiang Medical University, No. 137, Liyushan Road, Urumqi, 830011 China; 2 College of Basic Medicine of Xinjiang Medical University, Urumqi, 830054 China; 3 Department of Cardiology, First Affiliated Hospital of Xinjiang Medical University, Urumqi, 830011 China

**Keywords:** Cardiology, Diseases, Risk factors

## Abstract

PCSK9 plays a crucial role in lipid metabolism. This case–control study explored the associations of novel single nucleotide polymorphisms (SNPs) of the PCSK9 gene with coronary artery disease (CAD) (≥ 1 coronary artery stenosis ≥ 50%) and its risk factors in the Han population in Xinjiang, China. Four tag SNPs (rs11583680, rs2483205, rs2495477 and rs562556) of the PCSK9 gene were genotyped in 950 CAD patients and 1082 healthy controls. The distributions of genotypes in rs2483205 and rs562556 were significantly different between the groups (all *p* < 0.05). The TT genotype of rs2483205, GG genotype of rs562556, and their H4 (T-G) haplotype were associated with CAD [odds ratio (OR) 0.65, confidence interval (CI) 0.45–0.95, p = 0.024; 0.63, 0.45–0.90, p = 0.011; 0.50, 0.35–0.70, p < 0.001, respectively]. Additionally, the model (TT + CT vs. CC) of rs2483205 was associated with increased risk of obesity, and the G allele of rs562556 was associated with lower low-density lipoprotein cholesterol (LDL-C), blood glucose, body mass index (BMI), and mean platelet volume (MPV) (all p < 0.05). rs2483205, rs562556, and their H4 haplotype of the PCSK9 gene were associated with CAD. Additionally, rs2483205 is associated with obesity, and rs562556 is associated with LDL-C, blood glucose, BMI, and MPV.

## Introduction

Coronary artery disease (CAD) is the main cause of death worldwide. Genes are one of the main factors that affect the development of CAD, accounting for 40–60% of cases^1^. Currently, genes such as PCSK9 have been proven to significantly affect lipid metabolism and are associated with the development of CAD^2^.

The human PCSK9 gene, located on chromosome 1p32.3, is mainly expressed as an amino acid glycoprotein. As studies have reported, PCSK9 has effects on lipid metabolism mainly through the degradation of low-density lipoprotein (LDL) receptors^3^. The specific mechanisms are as follows: intracellularly, PCSK9 binds to the LDL receptor to promote lysosomal degradation of the receptor^4,5^, while extracellularly, PCSK9 conjoins the EGF-A domain of LDL receptors to prevent LDL receptors from recycling to the cell surface^5^. Dozens of studies have indicated that PCSK9 genetic mutations are associated with CAD, and more than 50 functional PCSK9 genetic mutations affect cholesterol levels in plasma^6,7^. Individuals with a loss-of-function (LOF) mutation in PCSK9 are likely to present with life-long low total cholesterol (TC) and LDL-C levels and a notably reduced incidence of CAD risk. In contrast, individuals with a gain-of-function (GOF) mutation probably manifest hypercholesterolemia and susceptibility to CAD^8,9^. According to studies, 2.6% of nonsense mutations in PCSK9 are responsible for a 28% decrease in LDL-C levels and an 88% decrease in the risk of CAD^9^. Some missense mutations of the PCSK9 gene also have a notable effect on plasma LDL-C levels and usually cause mild hypocholesterolaemia to protect against CAD^6^.

Previously, most studies on the PCSK9 gene were focused on several polymorphisms, such as E670G or R46L, and the findings suggested that the PCSK9 gene was significantly associated with LDL-C levels and CAD^10–12^. Recently, an increasing number of studies have implied that PCSK9 might affect CAD beyond its effects on lipids and that it also interacts with cardiovascular risk factors, such as insulin resistance^13,14^, inflammation^15^, platelet activation^16^, and thrombosis^17^. However, these multieffects of PCSK9 are still unclear, and whether there are novel genetic targets of PCSK9 that could be applied to therapy for atherosclerotic cardiovascular disease (ASCVD) with safer, more efficient, and pleiotropic effects requires further exploration.

Polymorphic sites of the PCSK9 gene vary in different regions and races. The polymorphisms of the PCSK9 gene in the Han population living in the northwestern part of China have not ever been reported. Moreover, finding novel polymorphisms and potential effects of the PCSK9 gene would contribute to finding potential therapeutic targets, determining its potential mechanisms, and expanding its applications in the clinic for CAD. Therefore, this study investigated whether there are novel polymorphisms of the PCSK9 gene associated with CAD and its risk factors in the Han population in Xinjiang, China.

## Results

### Clinical characteristics of participants

There were 950 CAD subjects (mean age of 58.68 ± 7.30 and 47.14% men) and 1082 controls (mean age of 58.68 ± 7.29 and 46.43% men) who were involved in the analysis. As Table 1 shows, comparing the groups, the baseline clinical characteristics of CAD patients were characterized by lower high-density lipoprotein (HDL) and higher body mass index (BMI), glucose, blood pressure, uric acid, triglycerides (TGs), and prevalences of smoking, hypertension, alcohol intake, diabetes, and statin therapy (all *p* < 0.05). However, the parameters of age, sex, LDL-C, and TC did not show any differences between the groups.

**Table 1:** Baseline characteristics of control subjects and patients with coronary heart disease.

Characteristics	Control (n = 1082)	CAD (n = 950)	*p* value
Age	56.67 ± 8.73	58.68 ± 7.29	0.981
Male gender, n (%)	514 (46.43)	436 (47.14)	0.746
Smoking, n (%)	327 (30.31)	453 (47.62)	< 0.001*
BMI, kg/m^2^	25.56 ± 4.02	26.37 ± 3.46	< 0.001*
SBP, mmHg	134.99 ± 22.61	150.50 ± 31.83	< 0.001*
DBP, mmHg	85.42 ± 16.63	92.10 ± 20.46	< 0.001*
Hypertension, n (%)	485 (45.07)	516 (54.3)	< 0.001*
Uric Acid, mmol/L	270.74 ± 85.22	310.77 ± 112.61	< 0.001*
Glucose, mmol/L	5.30 ± 1.90	6.23 ± 2.62	< 0.001*
Diabetes, n (%)	88 (8.13)	233 (24.53)	< 0.001*
TG, mmol/L	1.56 ± 1.07	2.03 ± 1.33	< 0.001*
TC, mmol/L	4.27 ± 0.93	4.38 ± 1.63	0.058
HDL-C, mmol/L	1.25 ± 0.47	0.98 ± 0.40	< 0.001*
LDL-C, mmol/L	2.66 ± 0.69	2.68 ± 0.98	0.591
Statin therapy, n (%)	124 (11.46)	238 (25.05)	< 0.001*

*BMI* body mass index, *SBP* systolic blood pressure, *DBP* diastolic blood pressure, *TC* total cholesterol, *TG* triglyceride, *LDL-C*low density lipoprotein-cholesterol, *HDL-C* high density lipoprotein-cholesterol.

*Compared with control group, *p*-values < 0.001.

### Distribution of genotypes of the polymorphisms of the PCSK9 gene between the CAD and control groups

Four SNPs of PCSK9 (rs11583680 C>T, rs2483205 C>T, rs2495477 A>G and rs562556 G>A) were genotyped in both the CAD and control groups. As Table 2 shows, the distribution of each genotype, genetic model, and allele of the four SNPs was separately examined in CAD and controls. Except for rs2495477 in the CAD group, all the genotype frequencies in both groups were in Hardy–Weinberg equilibrium (HWE) (*p* > 0.05). For rs2483205, the distributions of the CC, CT, and TT genotypes and of its recessive model (TT vs. CC + CT) were different between the two groups (*p* = 0.025 and *p* = 0.008, respectively). For rs562556, the distributions of the AA, AG, and GG genotypes; A and G alleles; and its dominant model (AA vs. GG + AG) were also significantly different between the two groups (*p* = 0.020, *p* = 0.005 and *p* = 0.006, respectively). However, comparing the control group, the distributions of genotypes, models, or alleles of rs11583680 and rs2495477 did not show any significant differences between the CAD and control groups (*p* = 0.294 and *p* = 0.342, respectively).

**Table 2:** Genotype and allele distributions in control subjects and CAD patients.

Variants	Model		Control, n (%) (n = 1082)	CAD, n (%) (n = 950)	CAD *p*-value (H-W)	Control *p*-value (H-W)	P-value∮
rs11583680(SNP1)	Genotypes	CC	882 (81.52)	750 (78.95)			
		CT	187 (17.28)	184 (19.37)			
		TT	13 (1.20)	16 (1.68)			0.294
	Dominant model	CC	882 (81.52)	750 (78.95)			
		TT + CT	200 (18.48)	200 (21.05)	0.231	0.389	0.146
	Recessive model	TT	13 (1.20)	16 (1.68)			
		CC + CT	1069 (98.80)	934 (98.32)			0.360
	Alleles	C allele	1951 (90.16)	1684 (88.63)			
		T allele	213 (9.84)	216 (11.37)			0.114
rs2483205 (SNP2)	Genotypes	CC	523 (48.34)	488 (51.37)			
		CT	456 (42.14)	402 (42.32)			
		TT	103 (9.25)	60 (6.32)			0.025**∮**
	Dominant model	CC	523 (48.34)	488 (51.37)			
		TT + CT	559 (51.66)	462 (48.63)	0.057	0.803	0.173
	Recessive model	TT	103 (9.52)	60 (6.32)			
		CC + CT	979 (90.48)	890 (93.68)			0.008**∮**
	Allele	C allele	1502 (69.41)	1298 (71.32)			
		T allele	662 (30.69)	522 (28.78)			0.189
rs2495477 (SNP3)	Genotypes	AA	502 (46.40)	463 (48.74)			
		AG	486 (44.92)	419 (44.11)			
		GG	94 (8.69)	68 (7.16)			0.342
	Dominant model	AA	502 (46.40)	463 (48.74)			
		GG + AG	580 (53.60)	487 (51.26)	0.041*	0.120	0.292
	Recessive model	GG	94 (8.69)	68 (7.16)			
		AA + AG	988 (91.31)	882 (92.84)			0.204
	Allele	A allele	1490 (68.9)	1345 (70.79)			
		G allele	674 (31.1)	555 (29.21)			0.180
rs562556 (SNP4)	Genotypes	AA	962 (88.91)	879 (92.53)			
		AG	118 (10.91)	70 (7.37)			
		GG	2 (0.18)	1 (0.11)			0.020**∮**
	Dominant model	AA	962 (88.91)	879 (92.53)			
		GG + AG	120 (11.09)	71 (7.47)	0.746	0.411	0.005**∮**
	Recessive model	GG	2 (0.18)	1 (0.11)			
		AA + AG	1080 (99.82)	949 (99.89)			0.641
	Allele	A allele	2042 (94.36)	1828 (96.21)			
		G allele	122 (5.54)	72 (3.79)			0.006**∮**

*CAD* coronary artery disease.

*p values < 0.05 for Hardy–Weinberg equilibrium in CAD patients and controls.

**∮***p* values < 0.05 for distribution frequency for genotypes and alleles of the 4 SNPs in the PCSK9 gene.

### Independent risk factors for CAD

To determine whether the polymorphisms of the PCSK9 gene were independent risk factors for CAD, we adjusted for confounding risk factors, including TGs, TC, HDL-C, LDL-C, and the prevalence of diabetes. No collinearity was existed in the regression models. We found that the recessive model (CC vs. TT + CT) of rs2483205 and the dominant model (AA vs. GG + AG) of rs562556 still showed a significant association with CAD. The TT genotype of rs2483205 indicated protective effects against CAD (OR = 0.65, 95% CI = 0.45–0.95, *p* = 0.024) (Table 3), and the GG genotype of rs562556 also exhibited a beneficial effect (OR = 0.63, 95% CI = 0.45–0.90, *p* = 0.011) (Table 4).

**Table 3:** Multiple logistic regression analysis for CAD patients and control subjects.

Risk factors	OR	95%CI	Wals	*p*
rs2483205(TT vs. CC + CT)	0.65	(0.45–0.95)	5.10	0.024*
Smoking	1.79	(1.46–2.19)	31.38	< 0.001*
Diabetes, n (%)	2.38	(1.55–3.64)	15.77	< 0.001*
TG, mmol/L	1.22	(1.13–1.32)	23.70	< 0.001*
HDL-C, mmol/L	0.22	(0.16–29)	102.88	< 0.001*
LDL, mmol/L	1.11	(0.96–1.29)	2.02	0.155

Adjust: smoking, diabetes, TG, HDL-C, and LDL-C.

*TG* triglyceride, *LDL-C* low density lipoprotein-cholesterol, *HDL-C* high density lipoprotein-cholesterol.

*p-values < 0.05.

**Table 4:** Multiple logistic regression analysis for CAD patients and control subjects.

Risk factors	OR	95%CI	wals	*p*
rs562556(GG + AG vs. AA)	0.63	(0.45–0.90)	6.42	0.011*
Smoking	1.76	(1.43–2.15)	29.40	< 0.001*
Diabetes, n (%)	2.40	(1.56–3.68)	16.07	< 0.001*
TG, mmol/L	1.22	(1.12–1.32)	22.71	< 0.001*
HDL-C, mmol/L	0.22	(0.16–0.29)	104.30	< 0.001*
LDL, mmol/L	1.11	(0.95–1.28)	1.79	0.180

Adjust: smoking, diabetes, TG, HDL-C, and LDL-C.

*TG* triglyceride, *LDL-C* low density lipoprotein-cholesterol, *HDL-C* high density lipoprotein-cholesterol.

*p-values < 0.05.

### Linkage disequilibrium (LD) analysis

LD analysis of the PCSK9 gene was performed (Supplementary Table S1). We identified that these four SNPs are located in the same haplotype block. Except for rs2483205 (SNP2) and rs2495477 (SNP3), all the r^2^ values of SNPs were below 0.5, which means that we could not construct haplotypes of SNP2 and SNP3 simultaneously. In addition, because the minor allele frequency (MAF) of SNP2 is larger than that of SNP3, we used rs11583680 (SNP1), SNP2, and rs562556 (SNP4) to construct the haplotypes. Furthermore, |D’| for SNP1-SNP2, SNP1–SNP3, and SNP1–SNP4 was < 0.5. Therefore, we did not use the SNP1 to construct haplotypes. Finally, SNP2 and SNP4 were used to construct haplotypes.

### Relationship between the haplotypes of the PCSK9 gene and CAD

As Table 5 shows, we established the haplotypes by combining SNP2 and SNP4. The distribution of haplotypes constructed by SNP2–SNP4 between the CAD and control groups was analysed. The haplotype distributions of C–G (H2) and T-G (H4) were significantly different between the two groups (*p* < 0.05). The frequencies of the H2 haplotype were significantly higher in the CAD group than in the control group (OR = 1.97, 95% CI: 1.013–3.842, *p* = 0.042). However, compared with the healthy control subjects, the frequencies of the H4 haplotype were significantly lower in patients with CAD (OR = 0.50, 95% CI: 0.35–0.70, *p* < 0.001).

**Table 5:** Haplotype analysis in patients with CAD and control subjects.

Haplotype	SNP2	SNP4	Control, n (%) (n = 1082)	CAD, n (%) (n = 950)	OR[95%CI]	*P* value
H1	C	A	1488.21 (0.69)	1354.26 (0.71)	1.13 [0.99–1.29]	0.082
H2	C	G	13.79 (0.01)	23.74 (0.01)	1.97 [1.01–3.84]	0.042*
H3	T	A	553.79 (0.26)	473.74 (0.25)	0.97 [0.84–1.11]	0.630
H4	T	G	108.21 (0.05)	48.26 (0.03)	0.49 [0.35–0.70]	< 0.001*

SNP2: rs2483205; SNP4: rs562556. *: *p* < 0.05.

### The relationship between the genotypes of polymorphisms of the PCSK9 gene and cardiovascular risk factors

To determine the effects of polymorphisms of the PCSK9 gene on CAD disease, we screened out the values of cardiovascular factors that exhibited differences among various genotypes of rs2483205 and rs562556 and explored their relationship. The results revealed that individuals with the TT genotype in rs2483205 showed decreased TC levels 0.43 mmol/L (9.91%) and increased incidence of obesity (4.28%). In the G allele group for rs562556, 0.30 mmol/L (11.07%) mean LDL-C levels and 0.52 mmol/L (9.06%) mean blood glucose levels were reduced, and 0.66 kg/m^2^ (2.49%) mean BMI values were increased than in the A allele group. No difference was observed in the values of HDL or TGs among genotypes in either rs2483205 or rs562556 (Table 6). According to the medians of TC (4.15 mmol/L), LDL-C (2.58 mmol/L), blood glucose (5.07 mmol/L), and BMI (25.78 kg/m^2^), all subjects were divided into high-value and low-value groups for each parameter. The results of univariate logistic regression analysis showed that genotypes with a T allele of rs2483205 were notably associated with a 30 percent increase in the incidence of obesity (Table 7), and the G allele of rs562556 associated with a 71 percent reduction in the incidence of high levels of LDL-C (≥ 2.58 mmol/L), 42 percent reduction in the incidence of high blood glucose(≥ 5.07 mmol/L), and 37 percent increase in the risk of high BMI (≥ 25.78 kg/m^2^) (Table 8).

**Table 6:** The comparison of CAD associated risk factors between different genotypes of rs2483205 and rs562556 polymorphisms.

Parameters	rs2483205					rs562556			
	CC genotype (n = 1010)	CT genotype (n = 858)	TT genotype (n = 163)	t /$\chi^{2}$	p value	A allele (n = 1814)	G allele (n = 188)	t /$\chi^{2}$	p value
LDL-C,mmol/L	2.70 ± 0.80	2.65 ± 0.88	2.61 ± 0.81	0.83	0.438	2.71 ± 0.99	2.41 ± 0.97	4.28	0.048*
TG,mmol/L	2.05 ± 2.77	2.06 ± 2.94	1.83 ± 1.33	0.45	0.636	1.61 ± 1.27	1.36 ± 0.80	4.25	0.400
TC,mmol/L	4.34 ± 1.77	4.62 ± 2.62	3.91 ± 1.20	3.34	0.036*	4.29 ± 0.94	4.17 ± 0.88	1.08	0.300
HDL,mmol/L	1.18 ± 0.67	1.15 ± 0.72	1.13 ± 0.42	0.49	0.615	1.16 ± 0.66	1.10 ± 0.41	0.14	0.230
Glucose,mmol/L	5.78 ± 2.27	5.63 ± 2.14	5.43 ± 2.03	2.15	0.116	5.74 ± 2.24	5.22 ± 1.75	8.86	0.003*
Diabetes, n (%)	65 (6.43)	62 (7.23)	9 (5.52)	0.86	0.650	128 (6.95)	8 (4.19)	2.12	0.171
BMI,kg/m^2^	25.76 ± 3.78	26.09 ± 3.83	26.34 ± 3.59	2.72	0.066	25.88 ± 3.77	26.54 ± 3.92	5.19	0.023*
Obesity, n (%)	242 (23.94)	250 (29.14)	46(28.22)	6.73	0.035*	477 (25.91)	61 (32.45)	4.83	0.089

*LDL-C* low density lipoprotein-cholesterol, *TG* triglyceride, *TC* total cholesterol, *HDL* high density lipoprotein-cholesterol, *BMI* body mass index.

*p-values < 0.05.

**Table 7:** Univariate logistic regression analysis of rs2483205 genotypes for cardiovascular risk factors.

rs2483205	CT + TT(n = 1021)	CC(n = 1010)	OR	95%CI	wals	*p*
TC(≥ 4.15 mmol/L)	400 (53.05%)	376 (48.76%)	0.84	0.69–1.03	2.80	0.095
Obesity(BMI ≥ 28 kg/m^2^)	296 (29.0%)	242 (23.9%)	1.30	1.06–1.58	6.65	0.010*

*OR* odds ratio, *CI* confidence interval, *TC* total cholesterol.

*p-values < 0.05.

**Table 8:** Univariate logistic regression analysis of rs562556 genotypes for cardiovascular risk factors.

rs562556	A allele(n = 1828)	G allele(194)		OR	95%CI	Wals	*p*
LDL-C (≥ 2.58 mmol/L)	1198 (65.07%)	63 (32.98%)	0.26		0.19–0.36	25.68	< 0.000*
Blood glucose (≥ 5.07 mmol/L)	1013 (55.02%)	79 (41.36%)	0.58		0.43–0.78	12.75	< 0.001*
BMI (≥ 25.78 kg/m^2^)	915 (49.70%)	110 (57.59%)	1.37		1.02–1.85	4.28	0.039*

*OR* odds ratio, *CI* confidence interval, *LDL-C* low density lipoprotein-cholesterol, *BMI* body mass index.

*p-values < 0.05.

### The relationship between PSCK9 genotypes and hemocyte parameters in subjects with CAD

To determine whether the polymorphisms of the PCSK9 gene have an effect on CAD-related hemocyte parameters in subjects with CAD, the values of hemocyte parameters were evaluated. The results showed that the red blood cell distribution width (RDW) was different among rs2483205 genotypes, and RDW, MPV, and activated partial thromboplastin time (APTT) exhibited a difference according to rs562556 alleles, and there was no difference in white blood cells (WBCs), neutrophils (NEs), vlymphocytes (LYs), monocytes (MOs), platelet count, or platelet distribution width (PDW) (Supplementary Table S2). Additionally, univariate logistic regression analysis showed that genotypes of rs2483205 had no relationship with hemocyte parameters (*p* > 0.05); however, the G allele of rs562556 was associated with two folds increase in the risk of high RDW (≥ 13.3%), and 43 percent reduction in the incidence of high MPV (≥ 10.2 fL) (Supplementary Table S3 and S4).

## Discussion

In this study, we found that two polymorphisms of rs562556, rs2483205, and their H4 haplotype of the PCSK9 gene were associated with CAD. Furthermore, the TT genotype of rs2483205 showed a 30 percent increase in the risk of obesity, and rs562556 polymorphisms were associated with high LDL-C, blood glucose, BMI, MPV, and RDW, which can increase the risk of CAD. These findings provide potential intervention targets and show the pleiotropic effects of PCSK9 in CAD.

A high LDL-C concentration is a pivotal risk factor for cardiovascular disease (CVD). Lower LDL-C levels are usually connected with a consistent and graded reduction in cardiovascular risk^18–22^. As a cornerstone and routine medicine in hyperlipidaemia therapy, statins could effectively lower LDL-C most of the time. However, due to potential side effects, some individuals’ poor response, and misinformation of statin therapy, many patients are reluctant to take statins or adhere to treatment^22,23^. In recent years, PCSK9 has been regarded as a promising therapeutic target to regulate cholesterol metabolism. Studies have indicated that even if patients are on maximum-dose statin treatment, PCSK9 inhibitors can still reduce plasma LDL-C levels by approximately 60%^23^. Recent studies revealed that PCSK9 inhibitors prevent CVD through mechanisms other than only reducing LDL-C levels. Several new effects of PCSK9 on diseases, such as insulin resistance and thrombus, have been revealed^13,17^.

A study observed no association between the rs562556 polymorphism and myocardial infarction but demonstrated an obvious relationship with LDL-C levels^24^. Two published studies presented significant associations between the rs562556 polymorphism and high levels of lipids in patients with hypercholesterolemia and polycystic ovary syndrome^25,26^. Recently, a meta-analysis study summarized that the G carriers of the rs562556 polymorphism had lower TC and LDL-C levels and relative risk than the noncarriers^27^. These findings are in agreement with our results that G carriers of the rs562556 polymorphism tended to have lower LDL-C levels. However, few studies have explored the relationship between the rs562556 polymorphism and CAD. Recently, a clinical study indicated that mutations of the rs562556 polymorphism were associated with both PCSK9 levels and arterial plaques^28^. According to these results, we suppose that the G allele of the rs562556 polymorphism is associated with a lower LDL-C level due to its low PCSK9 level, which contributes to protection against CAD.

Additionally, this study showed that the rs562556 polymorphism was associated with high blood glucose, BMI, MPV, and RDW, which might reveal new effects of the rs562556 polymorphism on CAD disease. The PCSK9 variants had effects on the risk of diabetes^13^. Depression was associated with the index of insulin resistance in obese individuals, which was partially mediated by plasma PCSK9 levels^14^, which would imply that the circulating level of PCSK9 could affect insulin resistance. Here, we observed that the mutation of the rs562556 polymorphism was associated with low blood glucose, which might be a result of carrying the G allele of the rs562556 polymorphism and having lower PCSK9 levels and lead to lower insulin resistance. MPV is both an indicator of platelet activation and a factor used to assess thrombolysis outcomes; moreover, it is related to the incidence of myocardial infarction^29,30,38,39^. In PCSK9 knockout mice, reduced platelet activation and reduced venous volume were observed. In the clinic, PCSK9 serum levels and residual platelet reactivity have shown a direct association in patients with acute coronary syndromes^31^. In the present study, we found that the rs562556 polymorphism was associated with MPV. Combined with the results of the above mentioned studies, we suppose that the rs562556 polymorphism probably suppresses platelet activation by decreasing plasma PCSK9 levels. However, one study showed that circulating PCSK9 levels are positively associated with the PLT count in CAD patients, while no correlation of PCSK9 with MPV was found^32^. This finding is contrary to our results, which would be due to differences in gene polymorphisms^33^. rs562556 was associated with RDW in CAD patients in this study, and their relationship has been less reported previously. RDW reflects erythrocyte size distribution, and previous studies have suggested that RDW accounted for almost 30% of the variance in RDW determined by more than 457 SNPs. In addition, RDW was significantly associated with LDL-C, HDL, TGs and inflammation. Therefore, the rs562556 polymorphism might affect RDW by lowering LDL-C levels. According to these results, we suppose that the G allele of rs562556 may decrease CAD risk by reducing circulating PCSK9 levels to decrease LDL-C, MPV, and RDW.

rs2483205 was screened out in two previous familial hypercholesterolemia studies. rs2483205 was an intronic variant in PCSK9, overlapping with 5 transcripts and regulating the promoter flanking region. A study showed that rs2483205 was associated with decreased LDL cholesterol concentration^34–36^. Here, we found that rs2483205 was independently associated with CAD in the Han population; however, it was not associated with LDL-C, which is different from the findings of a previous report. Additionally, the rs2483205 mutations were associated with an increased risk of obesity. Studies have reported that circulating PCSK9 levels mediate adipogenesis in visceral adipose tissue^37^ and are positively associated with cardiovascular disease risk in obese subjects^38^. Therefore, we hypothesize that rs2483205 may decrease CAD risk by modulating gene transcription and affecting TC levels, and it may be involved in adipogenesis by affecting PCSK9 levels, which enhances the risk of obesity.

Studies have indicated that variants in PCSK9 are independently associated with cardiovascular events through their effects on LDL-C levels^13^; however, as an important indicator of variants of the PCSK9 gene, whether plasma levels of PCSK9 could be a predictor of CVD is still unclear. Studies have shown that both the loss-of-function (LOF) R46L variant and gain-of-function D374Y mutation are associated with low plasma concentrations of PCSK9^39,40^. Low levels of PCSK9 decrease LDL-C levels by reducing degradation of the LDL receptor in the liver, which contributes to protection against CVD. Many studies have indicated that circulating PCSK9 levels are positively associated with cardiovascular disease and the risk of total cardiovascular (CV) events^38,41,42^. However, several studies have demonstrated contrary results: the plasma level of PCSK9 was not associated with CVD or adverse events^43,44^ and has less value to assess myocardial infarction risk in the general population than lipid measurements^45^. Cameron, J. et al. uncovered that defective LDLRs have an impact on circulating PCSK9^46^. Amy E Levenson et al. observed that among subjects with obesity and type 2 diabetes, higher circulating PCSK9 levels are observed in young women but not in young men^47^. These studies implied that although circulating PCSK9 levels play an important role in CVD, polymorphisms of PCSK9 would be a more reliable predictor for CVD than circulating PCSK9 levels, as circulating PCSK9 levels could be influenced by multiple factors.

Recently, a clinical study showed that circulating PCSK9 levels were independently associated with WBC, neutrophil, and lymphocyte counts, suggesting a potential interaction between PCSK9 and chronic inflammation in patients with CAD^48^. Carrying a PCSK9 LOF allele was associated with lower levels of pro-inflammatory cytokines in plasma of septic shock patients^49^, and circulating PCSK9 may mediate inflammation in the pathogenesis of atherosclerosis^15^. A study indicated that a weaker relationship was observed between PCSK9 and high-sensitivity C-reactive protein^44^. Here, the present study also examined WBCs, neutrophils, and lymphocytes, but they had no relationship with the rs2483205 or rs562556 polymorphisms in CAD subjects. These differences could be attributed to different effects of polymorphisms on inflammation.

The present study has several limitations. First, this is a case–control study, which provides less information about the relationship between PCSK9 polymorphisms and long-term prognosis or adverse CV events. Second, the sample size was small in this study. A prospective cohort study with a large sample size is required to further determine the relationship between PCSK9 polymorphisms and CAD disease and its prognosis. Third, information on circulating PCSK9 levels, which may partially explain the mechanisms of how the SNPs affecting CAD, was lacked in this study. Last, the effects and specific mechanisms of rs562556 or rs2483205 on CAD still require further basic and clinical research.

In conclusion, rs2483205, rs562556, and their H4 haplotype of the PCSK9 gene were associated with CAD. Additionally, rs2483205 is associated with obesity, and rs562556 is associated with high LDL-C, blood glucose, BMI, MPV, and RDW. The polymorphisms of rs562556 and rs2483205 in the PCSK9 gene would be a potential therapeutic target for the treatment of CAD and its risk factors.

## Methods

### Ethical approval of the study

All subjects gave us permission to proceed with DNA analysis and collecting relevant clinical data and signed informed consent forms. The Ethics Committee of the First Affiliated Hospital of Xinjiang Medical University reviewed the operating protocol and approved this study. All procedures were performed in accordance with the requirements of the Declaration of Helsinki.

### Subjects

In this study, we included 950 cases and 1082 healthy controls in the Han population who lived in Xinjiang, China. This study recruited patients who underwent angiography examination and were diagnosed with CAD at the First Affiliated Hospital of Xinjiang Medical University from 2008 to 2015. The control groups were randomly selected with age-matched participants from the cardiovascular risk survey (CRS) study, which has been reported previously^50,51^. In brief, this was a cross-sectional study that collected blood samples, demographic information, lifestyle data, and cardiovascular-related clinical characteristics from seven representative regions and multiethnic populations to explore the potential cardiovascular-related risk factors in the Xinjiang population of China. Individuals were excluded if they had a history of CAD. All the DNA samples of participants were extracted from the blood samples collected in EDTA-containing tubes.

The inclusion criteria were as follows: all the patients experienced symptoms of typical chest pain, and they underwent coronary angiography examination according to the guidelines^52^. CAD was defined as coronary angiography examination results demonstrating at least one coronary artery stenosis > 50%. The exclusion criteria were as follows: patients with valvular heart disease, non-ischaemic cardiomyopathy, heart failure, or congenital heart disease.

### Definition of cardiovascular risk factors

The standard of hypertension diagnosis was that, according to medical history and examination, individuals were diagnosed with hypertension before or at least 2 separate examination results showed systolic blood pressure (SBP) ≥ 140 mmHg or diastolic blood pressure (DBP) ≥ 90 mmHg in a resting state^53^. The diagnostic standard for diabetes was a history of diabetes, a glucose value > 11.1 mmol/L (200 mg/dl) at any time, or a glucose level > 7.0 mmol/L (126 mg/dl) in fasting plasma on 2 separate occasions. Smoking was defined as declaring regular tobacco use in the last 6 months. Subjects with a BMI ≥ 28 kg/m^2^ were defined as obesity.

### Routine blood test

Using freshly collected fasting peripheral blood samples, we performed routine biochemical variable testing. Lipid parameters were tested by the Dimension AR/AVL Clinical Chemistry System (DADE Bchring, Newark, NJ). Platelet parameters were examined by the CL TOP coagulation analyzer (Instrumentation Laboratory, USA). Hemocyte parameters were detected by the XN-2000 hematology analyzer (SYSMEX, Japan).

### Polymorphism selection and genotyping

We selected four tag SNPs by screening the 1000 Genomes Browser (https://www.ncbi.nlm.nih.gov/variation/tools/1000genomes/) and Haploview 4.2 software. Finally, rs11583680, rs2483205, rs2495477, and rs562556 were selected for the Chinese Han population. The cut-off of minor allele frequency (MAF) was set as > 0.05, and linkage disequilibrium (LD) patterns with r^2^ were set as > 0.8. rs11583680, rs2483205, rs2495477, and rs562556 belonged to the upstream transcript variant, intron variant, intron variant, and coding variant, respectively. The SNPs were genotyped using an improved multiplex ligation detection reaction (iMLDR) technique (Genesky Biotechnologies Inc., Shanghai, China). A blinded fashion was applied in genotyping in the absence of information on the patients' clinical data. Approximately 10% of the samples were genotyped twice to test the quality of genotyping results.

### Statistical analysis

According to data categories, we used different presentation and analysis methods. Continuous variables with a normal distribution are presented as the mean ± standard deviation (SD) and as the median in cases of a nonnormal distribution. The difference between two groups was examined by the independent Student’s *t*-test for variables with normal distribution, and variables were analysed by the Mann–Whitney U test when variables were nonnormally distributed. The chi-square test was applied to explore the differences in categorical variables. The independent association between polymorphisms and CAD was assessed by multiple logistic regression analysis. The collinearity of variables in regression models was analyzed by collinearity diagnostics. Hardy–Weinberg equilibrium (HWE) was analysed by the chi-square test by separately calculating the frequencies of genotypes in CAD and control subjects. Haplotype construction and LD tests were conducted by the SHEsis software platform^54^. A p-value < 0.05 represents statistical significance (2-tailed). SPSS version 22.0 software (SPSS, Inc., Chicago, IL) was used to perform all statistical analyses.

## Supplementary Information

Supplementary Information.
