# Supplementary material for: Polymorphisms of rs2483205 and rs562556 in the PCSK9 gene are associated with coronary artery disease and cardiovascular risk factors
Source: Sci Rep. 2021 Jun 1;11:11450. doi: 10.1038/s41598-021-90975-0 (PMC8169929; doi:10.1038/s41598-021-90975-0)
Supplement: Supplementary file 1 — Supplementary Information. [file 41598_2021_90975_MOESM1_ESM.docx]

**Polymorphisms of rs2483205 and rs562556 in the PCSK9 gene are associated with coronary artery disease and cardiovascular risk factors**

Min-Tao Gai, Dilare Adi, Xiao-Cui Chen, Fen Liu, Xiang Xie, Yi-Ning Yang, Xiao-Ming Gao, Xiang Ma, Zhen-Yan Fu, Yi-Tong Ma, Bang-dang Chen

Supplementary Table S1. Pairwise linkage disequilibrium for the four SNPs.

Supplementary Table S2. Comparison of cardiovascular risk factors between different genotypes of rs2483205 and rs562556 polymorphisms in CAD patients.

Supplementary Table S3. Univariate logistic regression analysis of rs2483205 genotypes for cardiovascular risk factors in CAD patients.

Supplementary Table S4. Univariate logistic regression analysis of rs562556 genotypes for hemocyte parameters in CAD patients

**Supplementary Table S1. Pairwise linkage disequilibrium for the four SNPs.**

|  |  | D'values | | | |
| --- | --- | --- | --- | --- | --- |
|  |  | SNP1 | SNP2 | SNP3 | SNP4 |
| r2 values | SNP1 |  | 0.44 | 0.41 | 0.08 |
|  | SNP2 | 0.06 |  | 0.82 | 0.72 |
|  | SNP3 | 0.05 | 0.63 |  | 0.78 |
|  | SNP4 | 0.00 | 0.06 | 0.07 |  |

|D'| above the diagonal and r^2^ below the diagonal. The shadowed portion indicates |D'|<0.5 and r^2^>0.5 SNP1-4 = rs11583680, rs2483205, rs2495477, rs562556.

|  | **rs2483205** | | | | |  | **rs562556** | | | |
| --- | --- | --- | --- | --- | --- | --- | --- | --- | --- | --- |
| **Parameters** | **CC genotype (n=488)** | **CT genotype**  **(n=402)** | **TT genotype (n=60)** | **t** | **p value** |  | **A allele**  **(n=914)** | **G allele**  **(n=36)** | **t** | **p value** |
| RDW,% | 13.50±1.48 | 13.64±2.05 | 14.42±3.45 | 5.63 | 0.004* |  | 13.56±1.86 | 14.24±2.50 | 7.51 | 0.006* |
| WBC,×10^9^/L | 7.19±2.11 | 7.19±1.91 | 7.22±1.82 | 0.01 | 0.993 |  | 7.21±2.03 | 6.95±1.83 | 0.99 | 0.324 |
| Neutrophil ,% | 59.85±10.84 | 59.74±10.59 | 58.56±12.29 | 0.34 | 0.710 |  | 59.71±10.88 | 59.92±10.19 | 0.339 | 0.881 |
| Lymphocyte ,% | 29.73±10.14 | 29.66±9.70 | 30.30±12.23 | 0.10 | 0.908 |  | 29.71±10.09 | 29.96±10.27 | 0.189 | 0.850 |
| Monocyte ,% | 7.50±2.31 | 7.19±2.22 | 7.33±2.05 | 1.83 | 0.162 |  | 7.36±2.27 | 7.34±2.12 | 0.06 | 0.953 |
| PLT,×10^9^/L | 210.63±62.05 | 208.88±59.52 | 213.16±62.88 | 0.16 | 0.857 |  | 210.22±61.02 | 208.40±61.68 | 0.23 | 0.820 |
| PDW,% | 16.68±2.21 | 16.81±2.15 | 17.06±3.56 | 0.80 | 0.449 |  | 16.76±2.26 | 16.72±2.75 | 0.13 | 0.900 |
| MPV,fL | 9.96±1.83 | 10.11±1.72 | 10.50±1.85 | 2.56 | 0.078 |  | 10.11±1.72 | 9.38±2.45 | 10.17 | 0.001* |
| APTT,s | 30.41±7.00 | 29.36±8.09 | 30.15±6.57 | 1.89 | 0.152 |  | 30.21±7.19 | 27.02±9.65 | 10.78 | 0.001* |

**Supplementary Table S2.** **The comparison of cardiovascular risk factors between different genotypes of rs2483205 and** **rs562556 polymorphisms in CAD**

Abbreviations: RDW, red blood cell distribution width;WBC, white blood cell; PLT, platelets; PDW, platelet distribution width; MPV, mean platelet volume; APTT, activated partial thromboplastin time. ***:** *p*-values <0.05.

**Supplementary Table S3. Univariate logistic regression analysis of rs2483205 genotypes for cardiovascular risk factors in CAD patients**

| **rs2483205** | **CT+TT(n=405)** | **CC(n=488)** | **OR** | **95%CI** | **wals** | ***p*** |
| --- | --- | --- | --- | --- | --- | --- |
| RDW (>13.3%) | 215(53.09%) | 251(51.43%) | 1.07 | 0.81-1.40 | 0.22 | 0.643 |

Abbreviations: OR, odds ratio; CI, confidence interval; RDW, red blood cell distribution width.

**Supplementary Table S4.** **Univariate logistic regression analysis of rs562556 genotypes for hemocyte in CAD patients**

| **rs562556** | **A allele(n=776)** | **G allele(65)** | **OR** | **95%CI** | **wals** | ***p*** |
| --- | --- | --- | --- | --- | --- | --- |
| RDW (>13.3%) | 396(50.97%) | 44(67.69%) | 2.02 | 1.18-3.45 | 6.51 | 0.011* |
| MPV (>10.2fl) | 418(53.87%) | 26(40.00%) | 0.57 | 0.34-0.94 | 4.87 | 0.027* |
| APTT (>31.00s) | 402(52.96%) | 26(41.27%) | 0.62 | 0.37-1.03 | 3.46 | 0.063 |

Abbreviations: OR, odds ratio; CI, confidence interval; RDW, red blood cell distribution width; MPV, mean platelet volume; APTT, activated partial thromboplastin time. ***:** *p*-values <0.05.
